# Usefulness of a Telemedicine Program in Refractory Older Congestive Heart Failure Patients

**DOI:** 10.3390/diseases6010010

**Published:** 2018-01-20

**Authors:** Emanuela Burdese, Marzia Testa, Pasquale Raucci, Cinzia Ferreri, Gabriele Giovannini, Enrico Lombardo, Enrico Avogadri, Mauro Feola

**Affiliations:** 1 Meditel s.r.l, 20100 Milano, Italy; emanuelaburdese@yahoo.it; 2 Department of Cardiovascular Rehabilitation-Heart Failure Unit Ospedale SS Trinità, Via Ospedale, 412045 Fossano (CN), Italy; marziatesta85@yahoo.it (M.T.); gabriele.giovannini@aslcn1.it (G.G.); enrico.lombardo@aslcn1.it (E.L.); enrico.avogadri@aslcn1.it (E.A.); 3 Clinical Engeneering, ASL CN1, 12100 Mondovi, Italy; pasquale.raucci@alice.it; 4 School of Geriatry, University of Turin, 10100 Turin, Italy; cinzia.ferreri@gmail.com; 5 Cardiology Division Ospedale Regina Montis Regalis, Strada del Rocchetto 99, 12084 Mondovi, Italy

**Keywords:** congestive heart failure, telemedicine, older adults

## Abstract

Background: Home telemonitoring is a modern and effective disease management model that is able to improve medical care, quality of life, and prognosis of chronically ill patients, and to reduce expenditure. The objective of this study was to evaluate the efficacy, costs, and patients’ and caregivers’ acceptance of our model of telemedicine in a high-risk chronic heart failure (CHF) older population. Methods: Patients with high risk/refractory CHF were included. In the case of alarm parameters’ modifications, a cardiologist decided to inform the emergency department (ED), the patient’s General Practioner, or to programme a clinical ambulatory control. Results: Forty-eight CHF patients (28 males; 58.3%), with a mean age of 80.4 ± 7.7 years, entered this clinical experience. During the 20-months follow-up, four patients dropped out from counselling (8.3%), ambulatory clinical control within-24 h was planned in 18% of patients, 11% of patients were admitted to an ED, and 18% were hospitalized. Thirteen patients (29.5%) died a cardiac death; hospital admissions for heart failure decreased during the year after the enrolment when compared to the year before (from 35 to 12 acute HF hospitalizations/year; *p* = 0.0001). Moreover, in these HF patients followed, accesses to an ED for an acute episode of HF decompensation reduced from 21/year to five/year (*p* = 0.0001). The economic expenditure, calculated for the year before and after the enrolment, reduced from 116.856 Euros to 40.065 Euros/year. Conclusions: A telemedicine surveillance in high-risk older CHF patients determines a continuous and active contact between patients/caregivers, the Heart Failure Clinic, and family physicians, permitting an early evaluation of signs and symptoms of acute decompensation.

## 1. Introduction

Heart failure (HF) is an increasing public health problem associated with high levels of morbidity, mortality, reduced quality of life [1], and high costs [2].

Chronic congestive heart failure (CHF) patients frequently undergo hospital readmission due to a worsening of hemodynamic conditions, poor therapy adherence, and incomplete clinical follow-up after discharge. The highest risk period for rehospitalisation is the first month after discharge [3] and up to 24% of patients should be readmitted [4]. Clinical surveillance (within the first week) seems to be effective in reducing hospital readmission. Many readmissions are known to be attributable to modifiable factors that can be addressed with high quality post-discharge care [5]. Multidisciplinary HF disease management programmes can reduce the hospitalisation rate, improve clinical outcomes [6], and significantly improve quality of life [7]. Home telemonitoring is a modern and effective disease management model able to improve medical care, quality of life, and the prognosis of chronically ill patients [8], and to reduce expenditure. A follow-up program tailored for refractory CHF older patients should be provided to avoid unnecessary rehospitalisation. Elderly patients with HF represent most of subjects (70%) admitted to hospital for acute cardiac decompensation; length of hospitalization usually lasts more than two weeks in geriatric wards and readmission is frequent [9]. Moreover, 40% of HF’s precipitating factors are removable and mortality (16%) and morbidity (45%) still remain high at six months after hospital discharge.

Recently, the OPTIMIZE–HF study [10], including more than 30,000 CHF patients discharged from 215 hospitals, described a shorter length of hospitalization (four days), but with a 21.3% rate of readmission to hospital within 30-days. This study also evidences that an early (one-week) outpatient clinical follow-up after discharge determines a lower risk of 30-day readmission. In the IN-HF Outcome, an Italian nationwide registry, the 30-day mortality after discharge for an acute episode of heart failure proved to be 2.8% and hospital readmission 6.2% [11]. An older age, longer in-hospital stay, necessity of inotropic agents, and worsening NYHA class identify discharged home HF patients that are at a higher risk of death or readmission. 

The objective of this study was to evaluate the efficacy and costs of our model of telemedicine in a high-risk CHF population discharged alive from hospital. In this clinical experience, we tested a new protocol that summed a model of nurse case management with disease management clinics in order to reduce hospital re-admissions in those patients. Moreover, we tried to analyse patients’ and caregivers’ acceptance of this home telemonitoring surveillance, which aimed to weight the customers’ satisfaction. 

## 2. Materials and Methods

We experienced the telemedicine project in some areas of ASLCN1 (Cuneo, Italy) and the Coordinating Centre was established to be the Cardiovascular Rehabilitation-Heart Failure Unit, SS Trinità Hospital in Fossano. Clinic protocol was approved by the hospital medical board. We performed a prospective observational study in high risk/refractory older CHF patients, discharged alive after an acute episode of cardiac decompensation, who were monitored by programmed home visit contacts. The aim of these visits was to discover alarm parameters and adherence to medical therapy. 

Consecutive patients included in the study were CHF patients and two or more of the following criteria should have been verified: age ≥70 years, left ventricular ejection fraction (LVEF) ≤30%, Brain Natriuretic Peptide (BNP) ≥250 pg/mL, NYHA (New York Heart Association) class ≥III, diastolic filling pattern grade ≥2, and a number of hospital admission ≥2 per year. Patients were included after discharge from the Cardiovascular Rehabilitation Unit (15%), Heart Failure Unit (57%), or after ambulatory visits (28%). 

The clinic protocol included: (a) a complete physical examination; (b) plasma determination of BNP and other laboratory tests (e.g., creatinine, haemoglobin); (c) a transthoracic echocardiogram; (d) an individualized HF disease education, emphasizing symptoms’ recognition and reporting; (e) an explanation of the telemedicine project; (f) signature of the informed consent; and (g) a discharge plan, including a medication list sent to the family physician.

The physical examination was performed by a cardiologist and focused on hemodynamic status and volume assessment. Patients were asked to bring all prescription medications and to observe educational/non-pharmacological advices on life-style (e.g., correct diet, quantity of water, and salt assumption, etc.). A cardiologist explained the telemedicine project to patients and family members or caregivers and collected a signature for informed consent. Family physicians were involved in the project and in patients’ enrolment and their consent was considered mandatory.

After discharging, the clinic protocol also included the following: (a) patient’s enrolment in the telemedicine project, (b) planned follow-up, and (c) a structured telephone support.

Telemonitoring of physiological parameters in patients with CHF may lead to an early identification of HF decompensation and may allow an early intervention before hospital readmission. Planned follow-up involved many participants, including a cardiologist as Project Director, a biologist and nurse as Project Coordinators, two cardiologists, specialized nurses and home care nurses, and finally family physicians. 

Planned follow-up consisted of a home visit within one-week after enrolment and then every two months or before according to clinical conditions. A visit might be required by patients or by their caregivers if symptoms of worsening heart failure have developed. At least every four months, a visit by a cardiologist was performed. 

**Figure 1:**
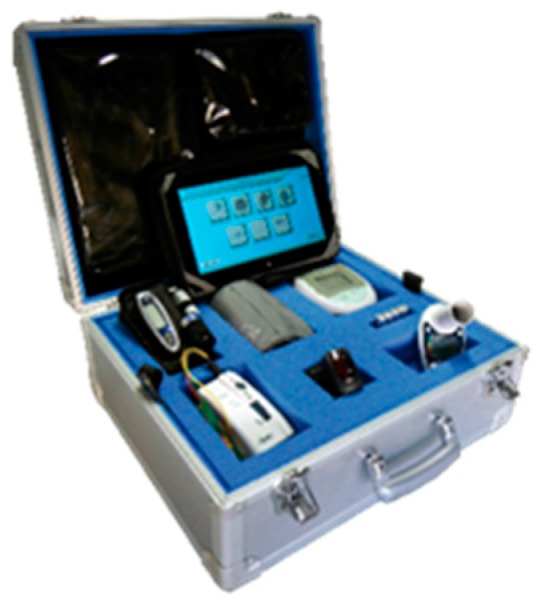
Nurse kit (KIT Lifechart Home_Meditel s.r.l) that consisted of a briefcase containing a tablet, a precision balance, an oxymeter, an electronic sphygmomanometer, and a 12-leads electrocardiograph (HeartView P-12/8).

The home-visit was performed by a home care nurse and consisted of a complete check of therapy adherence and general health status by compilation of a preformed electronic questionnaire in a portable table, regarding the presence of some alarms’ parameters which are reported later in the text. The home care nurse was equipped with a kit (KIT Lifechart Home_Meditel s.r.l) that consisted of a briefcase containing a tablet, a precision balance, an oxymeter (Nonin Medical INC, Plymouth MN USA), an electronic sphygmomanometer (Stabil Graph, GmbH Stolberg Germany), and a 12-leads electrocardiograph (HeartView P-12/8) (Aerotel Medical Systems Hazoref St-Holon, Holon, Israel) (Figure 1).

**Figure 2:**
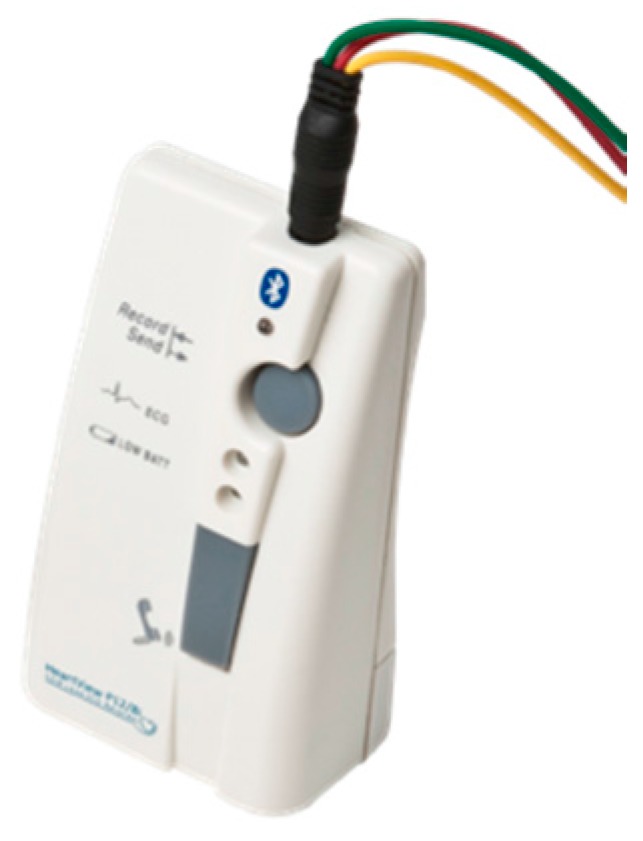
HearthView P-12/8 is a portable electrocardiograph capable of recording 12-leads electrocardiogram (ECG).

HearthView P-12/8 is a portable electrocardiograph capable of recording a 12-leads electrocardiogram (ECG) or, using a special selector, an 8-leads ECG. It is equipped with only three cables and four embedded electrodes. This device is extremely handy, absolutely reliable, and is of a small size (Figure 2). The tablet contained applications to manage the monitoring of patients. During the visit, an application was launched by the nurse and a folder with the patient’s personal data was opened. The tablet was able to give compact operative instructions to perform interactive measurements, and data were sent to the server through the Internet and inserted into a net platform (LifeChart Meditel) accessible by encrypted protected connection. During each visit, the final step was to compile the preformed electronic questionnaire.

In our CHF patients, five alarm parameters were monitored: (1) worsening of one or more NYHA class; (2) heart frequency <40/min or >110/min or the onset of a complete arrhythmic frequency; (3) angina pectoris or syncope; (4) increase of body weight >2–3 Kg with peripheral oedema; and (5) oliguria (<500 mL/day). In case of important modifications of the alarm parameters, the cardiologist decided to inform the emergency department (ED), the patient’s General Practitioner, or to programme an ambulatory control. 

Between one home visit and another, post-enrolment care consisted of structured telephone support, performed by specialized nurses educated for triage. Every fifteen days or every seven days, in more severe cases, patients were contacted by phone and their general health status was checked by the same preformed questionnaire of the home visit. If an alarm parameter was verified, the nurse decided to inform the cardiologist who programmed the correct assistance (ED, information to the patient’s family physician, or to programme a clinical control). 

The evaluation of BNP was measured by collecting a blood sample by venopuncture and this was immediately analyzed with the bedside Triage B type natriuretic fluorescence immunoassay (Biosite Diagnostics, La Jolla, CA, USA). The Triage Meter is used to measure BNP concentration by detecting a fluorescent emission that reproduces the amount of BNP in the blood. 

Echocardiograms were performed with a GE Vivid 7 Pro, according to the recommendations of the American Society of Echocardiograph [12]. Two-dimensional apical two- and four-chamber views were used for volume measurements; LVEF was calculated with a modified Simpson’s method using biplane apical (two- and four-chamber) views. Left ventricular systolic dysfunction was defined as an LVEF <50%. All echo examinations were performed by expert operators blinded to the results of the BNP assay; the intra-observer variability was found to be <5%. Medical therapy was normally assumed before the echocardiogram.

The 6MWT was performed at admission and discharge according to the ATS Statement of the American Thoracic Society [13]. CHF patients able to walk underwent 6MWT if they did not meet the exclusion criteria (unstable angina and myocardial infarction during the previous month; resting heart rate > 120; systolic blood pressure > 180 mmHg or diastolic blood pressure > 100 mmHg).

The Barthel Index was calculated both at admission and discharge. This is a scoring technique developed in 1965 and later modified by Granger et al. [14] that measures a patient’s performance in 10 activities of daily life. Items can be divided into a group related to self-care (feeding, grooming, bathing, dressing, bowel and bladder care, and toilet use) and a group related to mobility (ambulation, transfers, and stair climbing). The maximal score is 100, if five-point increments are used, indicating that the patient is fully independent in physical functioning. The lowest score is 0, representing a totally dependent bedridden state. 

For an evaluation of our telemedicine model’s acceptance, a satisfaction-preformed questionnaire was submitted to each patient. The quality questionnaire was created ad hoc by the Biomedical Engineer University of Turin (Turin, Italy) and aimed to explore the patients’/caregivers’ satisfaction of this telemonitoring protocol.

## 3. Statistical Analysis

Continuous variables were expressed as mean ± standard deviation (SD). Categorical variables were analysed using the chi-square test or Fisher’s exact test. For the comparisons between samples, we used the Mann-Whitney U-test and the Wilcoxon test. All probability values were two-tailed and differences were considered significant with a *p* value < 0.05. The 7.5 version of the SPSS software for Windows, release 12.0, SPSS Inc. Chicago, IL, USA was used. The cost-analysis related to this project considered the costs for medical cardiological in-hospital consultation, nurse visits at home, cardiological e-consultation of available parameters (ecg, blood pressure, etc.) after every nurse home-visit, and hospital reimbursement for CHF hospitalizations (according to the Italian DRG) and for ED examination, and was performed in a cross-over model, comparing the costs determined by the same patients included in the project the year before and the year after the beginning of the enrolment.

## 4. Results

Forty-eight CHF patients (28 males; 58.3%), with a mean age of 80.4 ± 7.7 years (range 53–93), entered this clinical experience between May 2014 and May 2015. Fifteen (31.2%) had an ischemic cardiomyopathy, 14 (29.2%) a valvular cardiomyopathy, and 19 (39.5%) another kind of cardiomypathy (e.g., hypertensive). The main characteristics of the population examined are reported in Table 1.

The population studied demonstrated to be quite old, and 80% had reduced left ventricular ejection fraction (LVEF), important activation of the neurohormonal asset (BNP), and impaired renal function. The Barthel index at admission depicted a mildly functionally independent population, although suffering from a high degree of dyspnea. Unfortunately, a six minute walking test was only performed in 14/44 patients able to undergo the test (mean 248.6 ± 88.4 m).

During the follow-up, which lasted 20 months, four patients dropped out from counselling (8.3%), whereas the other patients were visited at home for a total of 178 visits. Alarm parameters developed in 43% of patients; in particular, once in 25% of patients, twice in 16%, and finally four times in 2.3%. Among the 43 alarm parameters that emerged during the follow-up, syncope and angina pectoris never occurred, while disturbances in heart frequency developed in 3/43 cases, oliguria only in 2/43 cases, an increase of weight in 20/43 cases, and finally in 18/43 worsening of dyspnoea emerged. The weight of these different alarm parameters seemed to be quite different: in fact, while the increment of dyspnoea resulted in hospitalization/modified medical therapy in only 50% of patients, the development of oliguria was always correlated to hospitalization for worsening HF. 

A 24-h ambulatory clinical control was planned in 18% of patients, 11% of patients were admitted to an ED, and 18% were hospitalized for an acute HF decompensation. Thirteen patients died a cardiac death (29.5%) and hospital admissions for heart failure decreased during the year after the enrolment in the project, when compared to the year before (from 35 acute HF hospitalizations/year to 12 hospitalizations/year; *p* = 0.0001). Moreover, in these forty-four HF patients followed, accesses to an ED for suspect HF reduced from 21/year to five/year (*p* = 0.0001). The economic expenditure for these forty-four CHF patients reduced from 116.856 Euros to 40.065 Euros/year (Table 2). 

The quality questionnaire that explored patients’ and caregivers’ satisfaction was compiled by 27 patients (61.4%). The questionnaire (divided between low/medium/high grade of satisfaction) demonstrated a high degree of satisfaction in 74% of cases (the complete questionnaire is reported in Table 3).

## 5. Discussion

Home telemonitoring for complex HF patients opens new perspectives for the safe discharge of these chronically severe patients and shows potential benefits for patients’ quality of life and the containment of costs [15]. Many systematic reviews and meta-analyses document a 30–35% decrease in mortality and a 15–20% reduction in hospital admissions in home-telemonitored CHF patients [16,17,18,19] and, in some of them, a significant benefit in quality of life is also reported [16,18]. 

Another systematic review demonstrates a cost reduction from 1.6% to 68.3% from telemonitoring when compared to usual care, mainly attributed to reduced hospitalization expenditures and travel costs [20]. Takeda et al. [21], in a meta-analysis including 6000 patients coming from 25 trials, underlined that HF specialist nurse surveillance reduced CHF costs related to HF readmissions, total readmissions, and all-cause mortality. Therefore, in a budget impact analysis, telemedicine support in CHF patients seems to permit a progressive and linear decrease in costs dedicated to sanitary assistance. In the position paper of the Italian Cardiology Working group on Telemedicine, Brunetti et al. [22] dedicated a class IIB for the remote telemedicine monitoring in CHF in order to reduce hospital readmissions. According to the huge number of CHF patients discharged from our hospital, easy and practical prognostic parameters able to predict adverse outcomes are mandatory in order to correctly allocate our resources and to establish a tailored specific follow-up [23]. According to the QoL analysis, the meta-analysis of Knox et al. [24] demonstrated that telemedicine in HF was associated with a small significant increase in overall QoL (*p* = 0.001) and a positive trend (non-significant) for physical QoL. Very recently, Van Spall et al. [25] published a systematic review on the effectiveness of transitional care services in HF patients discharged alive from hospital, from which a proposed model of nurse case management (a combination of nurse home visits with structured telephone support) reduced hospital readmission, while disease management clinics (follow-up visits at hospital with a multidisciplinary HF management) ameliorated all-cause mortality in comparison to usual care.

In our experience, telemedicine surveillance in high risk older CHF patients (mean age 80.4 years) determines a continuous and active contact between patients/caregivers, the Heart Failure Clinic, and family physicians, permitting an early evaluation of signs and symptoms of acute decompensation, protecting from frequent hospital readmissions (during the 20-month follow-up), and reducing expenditure in the population examined before and after enrollment in the project. The quality questionnaire, moreover, clearly underlines how patients and caregivers interpreted the telemedicine model as useful and satisfactory, according to the positive results expressed in overall QoL. In the review of Bashushur et al. [26], based on 19 studies, the quality of life and costs of the telemedicine programs dedicated to HF patients seemed to gain satisfactory results, even if the differences in the results obtained might be related to different protocols, the length of follow-up, and selection of casistics.

In conclusion, a structured telemedicine program (nurse case management+disease management clinics) in older CHF patients seems to respond to the specific recommendations of the ESC Guidelines on HF [27] that suggested cardiologists consider the CHF patients >80 years old not only in terms of cardiovascular pathology, but also frailty and cognitive impairment. Those patients will benefit from closer contact with an HF specialist team and from more frequent and monitored visits. A home telemonitoring system, based on the cooperation among different medical/nurse figures, seemed to be able to reduce re-hospitalisations and ED accesses, reducing the expenditure for the follow-up of those patients (in comparison with the year before the enrolment). According to this preliminary data, the patients’/caregivers’ satisfaction of this telemonitoring program should be considered satisfactory.

## 6. Limitations of the Study

The main limitation of this study seems to be the limited number of CHF patients included, which reduced the possibility to differentiate the efficacy of this telemonitoring program according to the genesis of HF and the left ventricular function. Another important limitation was the influence and participation of GPs in the project. In fact, when the program started, the participation of GPs was limited to authorization of the inclusion of the CHF subject in the telemonitoring project. Nevertheless, GPs nowadays might actively include CHF patients in the project and follow, according to their personal access in the web page of telemedicine, the clinical conditions and parameters of their patients. Moreover, a control group, randomized to usual care, might be helpful in the comparison of the expenditure in the two different strategies. However, the head-to-head comparison between the economic costs in the 12-months period before the enrolment in the project with the one-year period after the enrolment (cross-over) seems to be a correct method to calculate the total amount of the expenditure required to follow those patients. 

## Figures and Tables

**Table 1:** Main characteristics of the population examined.

	*n* = 48
Age	80.4 ± 7.7 (range 53–93)
Males	28 (58.3%)
Ischemic CMP	15
Valvular CMP	14
Other CMP	19
SR/AF	20/28
Creatinine (mg/dL)	1.6 ± 0.6
Haemoglobin (g/dL)	11.7± 1.0
Sodium (mEq/L)	139.6 ± 2.9
LVEF [%] in patients with preserved EF (EF ≥ 55%) (*n* = 11)	58.9± 4.9
LVEF [%] in patients with impaired EF (EF < 55%) (*n* = 37)	33.2 ± 10.7
NYHA at enrolment	3.2 ± 0.8
BNP at enrollment (pg/mL)	1082.9 ± 1146.6
6MWT at enrollment (m) (*n* = 14)	248.6 ± 88.4
Barthel Index at enrolment	77.9 ± 23.7
Alarm parameters (%) (*n* = 44)	43
24 h Ambulatory visit (%) (*n* = 44)	18
ED visit (%) (*n* = 44)	11
Hospital admission (%) (*n* = 44)	18
Cardiovascular death (*n* = 44)	13 (29.5%)
Lost at FU	4 (8.3%)

CMP: cardiomyopathy; SR/AF: sinus rhythm/atrial fibrillation; LVEF: left ventricular ejection fraction; NYHA: New York Heart Association; BNP: brain natriuretic peptide; 6MWT: six minute walking test; ED: emergency department; FU: follow-up.

**Table 2:** Costs and readmissions intra-patient’s comparison between the year before and the year after the enrolment in Telemedicine program (*n* = 44 pt).

	Costs (€/Year)	ED Admissions (*n*°/Year)	30-Day Readmission (*n*°)
Before Telemedicine enrolment	116.856	21 (47.7%)	35 (21.3%)
After Telemedicine enrollment	40.065	5 (11.4%)	12 (6.2%)

ED: emergency department. *n*° = number

**Table 3:** Satisfaction questionnaire (*n* = 27 pt).

Satisfaction Questionnaire	(*n*° of Patients)
How to assess the number of visits received at home?	0–40% (4)
	40–80% (23)
	80–100% (0)
What is the possibility of carrying out checks at home instead of in hospital?	0–40% (0)
	40–80% (10)
	80–100% (17)
Do you prefer to carry out checks at home instead of in hospital?	0–40% (1)
	40–80% (11)
	80–100% (15)
Is the presence of a nurse at home helpful?	0–40% (0)
	40–80% (7)
	80–100% (20)
Were caregivers satisfied with the visit at home instead of in hospital?	0–40% (0)
	40–80% (20)
	80–100% (7)
Have additional check-ups required by the doctor been helpful?	0–40% (0)
	40–80% (21)
	80–100% (6)
Would you recommend to a friend, if needed, this telemedicine service?	0–40% (0)
	40–80% (7)
	80–100% (20)

Legend: 0–40% poorly satisfied; 40–80% medium satisfied; 80–100% highly satisfied.
